# Supplementary material for: Analysis of PPARγ Signaling Activity in Psoriasis
Source: Int J Mol Sci. 2021 Aug 10;22(16):8603. doi: 10.3390/ijms22168603 (PMC8395241; doi:10.3390/ijms22168603)
Supplement: Supplementary file 1 [file ijms-22-08603-s001.zip › Supplemental materials_Analysis of PPARg signaling activity in psoriasis/Pathway models/Models images and html files/PPARG expressed in cells assotiated with psoriasis/225630.html]

eosinophil


# CellType eosinophil

|  |  |
| --- | --- |
| URN | urn:agi-ncimcelltype:C0014467 |
| Total Entities | 52 |
| Connectivity | 4096 |
| Name | eosinophil |

---

|  |  |
| --- | --- |
| ChildConcepts | eosinophil function |
|  | eosinophil adhesion |
|  | eosinophil migration |
|  | eosinophil differentiation |
|  | eosinophil chemotaxis |
|  | eosinophil infiltration |
|  | eosinophil formation |
|  | eosinophil phenotype |
|  | eosinophil apoptosis |
|  | eosinophil transendothelial migration |
|  | eosinophil phagocytosis |
|  | eosinophil survival |
|  | eosinophil polarity |
|  | eosinophil homeostasis |
|  | eosinophil motion |
|  | eosinophil homing |
|  | eosinophil proliferation |
|  | eosinophil localization |
|  | eosinophil interaction |
|  | eosinophil development |
|  | eosinophil renewal |
|  | eosinophil extravasation |
|  | eosinophil death |
|  | eosinophil sequestration |
|  | eosinophil contact |
|  | eosinophil growth |
|  | eosinophil rolling |
|  | eosinophil spreading |
|  | eosinophil motility |
|  | eosinophil population |
|  | eosinophil tethering |
|  | eosinophil priming |
|  | eosinophil cytotoxicity |
|  | eosinophil fate commitment |
|  | eosinophil aggregation |
|  | eosinophil diapedesis |
|  | eosinophil mediated cytotoxicity |
|  | eosinophil selection |
|  | eosinophil lifespan |
|  | eosinophil transepithelial migration |
|  | eosinophil invasion |
|  | eosinophil ratio |
|  | eosinophil fate |
|  | eosinophil distribution |
|  | eosinophil size |
|  | eosinophil volume |
|  | eosinophil recognition |
|  | eosinophil swelling |
|  | eosinophil disruption |
|  | eosinophil repertoire |
|  | eosinophil macrophage differentiation |
|  | eosinophil compartmentalization |

---

|  |  |
| --- | --- |
| Pathway | C5AR1 -> Expression Targets in Lymphoid System and Blood |
|  | PPARG expressed in cells assotiated with psoriasis |
|  | 3\_2\_Regulation of airway surface liquid (ASL) thickness |
|  | 2\_3\_Eosinophilia and neutrophilia in asthma: Neutrophil chemotaxis and activation |

---

|  |  |
| --- | --- |
| MedScan ID | 10000127 |

---

|  |  |
| --- | --- |
| Alias | eosinophil granulocytes |
|  | eosinophilic |
|  | acidophilic leukocyte |
|  | eosinophil leukocytes |
|  | eosinophylic granulocyte |
|  | eosinophil leukocyte |
|  | acidophil leukocyte |
|  | acidophilic granulocyte |
|  | acidophil leukocytes |
|  | eosinophyl leukocyte |
|  | eosinocytes |
|  | acidophilic leukocytes |
|  | eosinophile granulocyte |
|  | eosinocytic |
|  | eosinophils |
|  | eosinophilic granulocyte |
|  | eosinophilic leukocyte |
|  | acidophil granulocyte |
|  | eosinophile granulocytes |
|  | eosinophil granulocyte |
|  | eosinophil |
|  | eosinocyte |
|  | eosinophilic leukocytes |
|  | eosinophilic granulocytes |

---

|  |  |
| --- | --- |
| NCIm ID | C0014467 |

---
